# *Helicobacter cinaedi* Infection of Abdominal Aortic Aneurysm, Japan

**DOI:** 10.3201/eid2011.140440

**Published:** 2014-11

**Authors:** Risako Kakuta, Hisakazu Yano, Hajime Kanamori, Takuya Shimizu, Yoshiaki Gu, Masumitsu Hatta, Tetsuji Aoyagi, Shiro Endo, Shinya Inomata, Chihiro Oe, Koichi Tokuda, Daiki Ozawa, Hitoshi Goto, Yukio Katori, Mitsuo Kaku

**Affiliations:** Tohoku University Graduate School of Medicine, Sendai, Japan

**Keywords:** Infected abdominal aortic aneurysm, IAAA, *Helicobacter cinaedi*, bacteria, 16S ribosomal RNA gene, antibiotic, antibacterial, antimicrobial, immunocompromised, Japan

**To the Editor:** Infected abdominal aortic aneurysm (IAAA) is uncommon, but life-threatening; the mortality rate ranges from 25% to30% (*1*,*2*). Identification of the pathogen is essential for diagnosis and treatment. Previous studies have shown that species of the genera *Salmonella*, *Staphylococcus*, and *Streptococcus* are the most common pathogens associated with IAAA, but a causative organism is not identified in 14%–40% of patients (*1*,*2*). *Helicobacter cinaedi* has mainly been isolated from immunocompromised patients with bacteremia, cellulitis, and septic arthritis (*3*,*4*). Here, we report 3 cases of IAAA caused by *H. cinaedi* detected by 16S ribosomal RNA (16S rRNA) gene analysis.

The 3 patients (case-patients 1–3) were referred to Tohoku University Hospital, Sendai, Japan, for surgical treatment of IAAA in 2013. None had a history of disease known to cause immunodeficiency. Because their abdominal aneurysms enlarged rapidly, all 3 patients underwent resection of the aneurysm and extensive local debridement and irrigation. Histopathologic examination of the surgical specimens revealed severe atherosclerosis and inflammation, consistent with a diagnosis of IAAA. For each case-patient, blood culture (BacT/ALERT; bioMérieux Industry, Tokyo, Japan) was negative, as was culture of surgically removed tissue on HK semisolid agar (Kyokuto Pharmaceutical Industrial Co., Ltd., Tokyo, Japan) at 35°C under aerobic conditions for 7 days for enrichment of microorganisms, and on chocolate agar at 35°C under 5% CO_2_ for 48 h. We then used 16S rRNA gene analysis to identify a pathogen. We extracted DNA from resected tissues using a QIAamp DNA Mini kit (QIAGEN K.K., Tokyo, Japan), amplified it using PCR, and sequenced it using universal primers for 16S rRNA (*5*). We used the EzTaxon-e Database for sequence analysis (http://eztaxon-e.ezbiocloud.net/), which revealed that the 16S rRNA gene sequence of bacteria in the aneurysmal tissues was identical to that of *H. cinaedi*. 

For case-patient 3, we cultured microaerophilic tissue at 35°C using Trypticase Soy Agar II with 5% sheep blood (Kyokuto Pharmaceutical Industrial Co.) and an Anaero Pouch-MicroAero (Mitsubishi Gas Chemical Co., Inc., Tokyo, Japan) to detect *H. cinaedi*. We observed bacterial colonies, after Gram staining, which showed gram-negative spiral rods. By 16S rRNA gene analysis, we confirmed that the isolate was *H. cinaedi*. 

For each of the 3 case-patients, species identification was further confirmed by sequence analysis of 23S ribosomal RNA (23S rRNA) (DNA Data Bank of Japan: http://blast.ddbj.nig.ac.jp/blastn?lang = ja) and amplification of the *gyrB* gene region that is specific to *H. cinaedi* (*6*,*7*). In samples from the 3 patients, there were mutations of the 23S rRNA gene and amino acid substitutions in GyrA related to macrolide and fluoroquinolone resistance, respectively (*6*,*8*). After identifying the pathogen, we selected antimicrobial agents based on the reported drug susceptibility profile of *H. cinaedi* (*6*,*8*). The patients survived and are being followed up as outpatients. Clinical and molecular characteristics of the 3 cases of IAAA with *H. cinaedi* infection are shown in the Table.

**Table:** Clinical characteristics of 3 patients with *Helicobacter cinaedi–*infected abdominal aortic aneurysms and molecular characteristics of isolates, Japan *

Characteristic	Case-patient 1	Case-patient 2	Case-patient 3
Age, y/sex	64/M	59/M	62/M
Underlying diseases	Hypertension, hyperlipidemia	None	History of myocardial infarction
Risk factors for infection	None	None	None
Clinical signs and symptoms before surgery	Fever, back pain	Fever, abdominal pain	Low back pain
CT results			
Site of aneurysm	Infrarenal abdominal, bilateral common iliac, internal iliac, L femoral, aortic arch†	Infrarenal abdominal, bilateral common iliac	Infrarenal abdominal
Inflammatory findings around aneurysms	+	+	+
Maximum leukocyte count/μL)/C-reactive protein, mg/dL before operation	10,600/25.3	9,100/6.05	7,050/ 5.29
Surgical management	In situ grafting	In situ grafting	In situ grafting
Microbiological diagnosis			
Blood culture	–	–	–
Tissue culture	–	–	+‡
rRNA gene sequence similarity, %§			
16S	99.8	99.6	99.6
23S	99.8	99.8	99.8
Amplification of *gyrB* specific to *H. cinaedi*	+	+	+
Aneurysms in which *H. cinaedi* was identified	Infrarenal abdominal, L common iliac, R internal iliac, L femoral	Infrarenal abdominal	Infrarenal abdominal
MLST	ST15 (CC7)	ST10 (CC9)	ST10 (CC9)
Mutation of 23S rRNA gene and amino acid substitutions in GyrA	2018 A→G and T84I D88G	2018 A→G and T84I	2018 A→G and T84I
Antimicrobial therapy dosage and duration			
Before admission	Ceftriaxone, 2 g/d, and levofloxacin, 500 mg/d, for 2 d	Piperacillin/tazobactam, 4.5 g/d for 12 d; faropenem sodium hydrate, 600 mg/d for 10 d	Oral antimicrobial agent, 4 d
After admission	Doripenem, 1.5 g/d for 22 d, and vancomycin, 3.0 g/d, for 14 d	Piperacillin/tazobactam, 4.5 g/d for 28 d	Doripenem, 1.5 g/d for 28 d
After identification of pathogen	Sulbactam/ampicillin, 3.0 g/d, and minocycline, 100 mg/d for 25 d	Continuation of piperacillin/tazobactam	Continuation of doripenem
At discharge	Oral amoxicillin, 1,500 mg/d, and minocycline, 200 mg/d, until follow-up visit	Oral amoxicillin, 1,500 mg/d, and minocycline, 200 mg/d, until follow-up visit	Oral amoxicillin, 1,500 mg/d, and minocycline, 200 mg/d, until follow-up visit
Postoperative complications	None	None	None
Outcome	Survived	Survived	Survived

*CT, computed tomography; +, positive; –, negative; L, left; R, right; MLST, multilocus sequence typing; ST, sequence type; CC, clonal complex; A, adenine; G, guanine; T, threonine; I, isoleucine; D, aspartic acid; G, glycine. †Aortic arch was replaced 5 weeks after the abdominal operation. ‡Species unidentifiable under microaerophilic conditions. §Compared with the type strain of *H. cinaedi* (CCUG 18818).

Although the high negative culture rate for pathogens causing IAAA had been explained by prolonged preoperative antimicrobial drug therapy (*2*), another possibility is that *H. cinaedi* may be a causative organism. Earlier research has suggested that *H. cinaedi* infections can remain undiagnosed or be incorrectly diagnosed because of difficulty in isolating this microorganism (*9*). *H. cinaedi* grows slowly under microaerophilic conditions, but no current standard laboratory methods result in a diagnosis of this pathogen (*6*,*7*,*9*). We isolated *H. cinaedi* from surgically removed tissue from case-patient 3 by microaerophilic culture after taking this pathogen into consideration. For diagnosis of *H. cinaedi* infections, methods leading to accurate identification by clinical microbiological laboratories are needed. Currently, *H. cinaedi* is identified by molecular analysis of the 16S rRNA gene (*6*,*7*,*10*). In addition, matrix-assisted laser desorption/ionization–time-of-flight mass spectrometry (MALDI-TOF MS) (*10*), may become a useful tool for this purpose. 

Standard breakpoints of antimicrobial drugs for *H. cinaedi* have not been defined, but all isolates in this study had mutations that indicated resistance to macrolides and fluoroquinolones. For adequate treatment for *H. cinaedi* infections, guidelines for selection of antimicrobial drugs and surveillance of its antimicrobial susceptibility profile are required.

During November 2012–November 2013, 8 patients underwent their first operation for IAAA at the university hospital. We used 16S rRNA gene analysis of surgical tissues and culture of blood and tissue specimens to detect pathogens (data not shown). Identification of *H. cinaedi* in 3 of 8 patients suggests that it could be a prevalent pathogen related to IAAA. Taking such information into consideration could affect the prognosis of many patients. Accordingly, tissue should be cultured while considering *H. cinaedi* infection in patients with IAAA. *H. cinaedi* colonizes the gastrointestinal tract, and bacterial translocation may lead to bacteremia associated with mucosal damage (*4*). However, the route of transmission and reason most *H. cinaedi* infections have been reported in Japan are unclear. To clarify the relationship between *H. cinaedi* and IAAA, further clinical and epidemiologic studies are needed. Meanwhile, we recommend clinical consideration of *H. cinaedi* infection, use of appropriate laboratory procedures to identify cases, and development of treatment guidelines.
